# Editorial: Heterogeneity in breast cancer: clinical and therapeutic implications

**DOI:** 10.3389/fonc.2024.1321654

**Published:** 2024-02-26

**Authors:** Francesca Carlino, Cinzia Solinas, Michele Orditura, Maria Dezia Bisceglia, Benedetta Pellegrino, Anna Diana

**Affiliations:** ^1^ Medical Oncology Unit, Ospedale Ave Gratia Plena, San Felice a Cancello, Caserta, Italy; ^2^ Medical Oncology, Azienda Ospedaliera Universitaria (A. O. U.) Cagliari Policlinico Duilio Casula di Monserrato, Cagliari, Italy; ^3^ Medical Oncology Unit, Azienda Ospedaliera di Rilievo Nazionale (A.O. R. N.) Sant’Anna e San Sebastiano, Caserta, Italy; ^4^ Department of Pharmacy, Azienda Ospedaliera di Rilievo Nazionale (A.O. R. N.) Sant’Anna e San Sebastiano, Caserta, Italy; ^5^ Medical Oncology and Breast Unit, University Hospital of Parma, Parma, Italy; ^6^ Medical Oncology Unit, Ospedale del Mare, Naples, Italy

**Keywords:** breast cancer, heterogeneity, molecular mechanisms, drug resistance, emerging technologies

Breast cancer (BC) is a complex disease with high intratumoral and intertumoral heterogeneity. Such heterogeneity plays a critical role in treatment response, therapeutic failure, and disease outcome (1). Despite significant advances in early detection and therapy, BC remains the leading cause of cancer-related death in women worldwide (2). While clinicians and researchers are actively engaged in identifying the optimal treatment strategy, the limited understanding of the molecular mechanism of BC heterogeneity in the context of drug resistance and disease recurrence represents one of the major challenges in current BC research. To address this issue, there is a growing interest in developing innovative methods to better understand the mechanisms underlying BC heterogeneity in order to facilitate effective diagnosis and provide tailored treatment.

The Research Topic entitled “*Heterogeneity in Breast Cancer: Clinical and Therapeutic Implications*” includes 16 research articles, 1 review, 1 network meta-analysis, and 1 case report that address various aspects of heterogeneity in BC disease: histologic and immunohistochemical characteristics, clinical manifestations, radiomic features, surgical and medical approaches, treatment responses, implications of DNA repair gene alterations and treatment adherence. Below are the main topics covered in the various articles.

Conventional imaging techniques, such as mammography, ultrasound, and magnetic resonance imaging (MRI) are effective tools for measuring heterogeneity in BC patients. Several studies have demonstrated that specific imaging-related features such as mass lesion shape, margin characteristics, T2 signal intensity, and contrast enhancement dynamics, reflect the distinct molecular subtypes of breast tumors.

Moreover, in order to improve current prognostic models and treatment planning, radiomics, a non-invasive approach that combines quantitative features extracted from medical imaging with genomic biosignatures, has emerged in recent years as a strategy to study BC heterogeneity (3).

Phyllodes tumors are uncommon neoplasms that exhibit both epithelial and mesenchymal characteristics, resembling fibroadenomas in terms of their histological appearance. These tumors can range in morphological presentation from benign to malignant. When assessed by conventional MRI, it can be challenging to distinguish between the features of benign, borderline, and malignant phyllodes tumors due to their overlapping characteristics (4). In their retrospective study, Fang et al. demonstrated that the apparent diffusion coefficient (ADC) value, a parameter derived from diffusion-weighted imaging (DWI), offers quantitative information with the ability to differentiate between phyllodes tumors, fibroadenomas, and breast neoplasms and to provide a classification of phyllodes tumors.

The integration of histological, clinicopathological, and molecular information, in addition to individual patient characteristics and preferences, is essential to establishing the optimal therapeutic pathway for a patient. 

Surrogate classification of BC subtypes based on biological markers such as estrogen receptor (ER), progesterone receptor (PR), human epidermal growth factor receptor 2 (HER2), and Ki67 expression levels accurately predict clinical characteristics of recurrence patterns and disease-free survival. Several studies on BC have revealed that single Progesterone Receptor (sPR) expression is associated with more aggressive behavior in early-stage BC, resembling the characteristics of triple-negative breast cancer (TNBC) (5). Luo et al. conducted a retrospective analysis involving a large cohort of 10,877 metastatic BC patients to understand the behavior and prognosis of sPR-positive and TNBC patients with advanced disease. The study results suggest that, as in the early stage, even in the advanced or metastatic setting, sPR-positive and TNBC patients show similar biological behavior supporting chemotherapy as the preferred treatment option for these subtypes.

Triple-positive breast cancer (TPBC), characterized by positivity for HER2, ER, and PR, is a rare subtype displaying features linked to a less favorable prognosis compared to other Luminal B-like BC (6).

To improve risk assessment, Geng et al. conducted a retrospective analysis of data from the Fourth Military Medical University Affiliated Xijing Hospital and the SEER database. The study identified several independent risk factors affecting the prognosis of TPBC patients, including age, chemotherapy, radiotherapy, TNM stage, and the type of surgery. These prognostic variables were then utilized to construct a nomogram designed to predict the 3-year and 5-year overall survival rates of TPBC patients. Nomograms are statistical prognostic models that are particularly useful for individualizing the clinical decision-making process, especially in the case of rare tumor types, and provide an easier estimation of the probability of a specific event than that with traditional evaluation methods (7). In particular, this nomogram serves as a valuable tool for clinicians to estimate and communicate the likelihood of survival outcomes based on individual patient characteristics and treatment modalities.

Approximately half of breast cancers, traditionally classified as HER2 negative exhibit low levels of HER2 expression, identified by an immunohistochemical (IHC) score of 1+ or 2+ with negative *in situ* hybridization. Retrospective data suggests that HER2-low BC does not represent a distinct subtype in terms of biological characteristics. Nevertheless, the prognostic impact of HER2-low expression BC remains controversial (8).

In a meta-analysis of 14 studies involving 52106 patients Wei et al. found that among early-stage, HER2-low-expressing BC patients, OS was better in the overall population and the hormone receptor-positive and TNBC subgroups. Notably, favorable DFS and RFS were observed in both the overall population and the hormone receptor-positive subgroup.

Since HER2-low breast cancer is highly unstable during disease progression, Shang et al. explored the evolution of HER2 expression in primary breast cancer and residual tumors after neoadjuvant therapy in 775 patients with pathological non-pCR breast cancer after preoperative therapy. HER2-low-expressing breast cancers accounted for just over half (59.61%) of the total HER2-negative cohort, with the proportion of HER2-low cases in breast cancer samples with residual tumors after neoadjuvant therapy being lower than in BC primaries. This discrepancy was primarily attributed to the phenomenon of HER2-low cases switching to HER2-zero status. Specifically, approximately 17% of patients with HER2-low primary BC experienced a transition to HER2-zero status following neoadjuvant therapy. In contrast, approximately 38% of patients initially identified as HER2-zero in the primary tumor shifted to HER2-low, providing additional evidence of the instability associated with HER2-low expression. This study confirmed the correlation between HER2-low and HR status but also demonstrated a correlation with AR status. These findings underscore the importance of re-evaluating HER2 status in BC patients following neoadjuvant therapy. This approach expands the range of treatment options available to patients. However, whether HER2-low BC can be definitively classified as a new subtype requires further confirmation through additional studies.

The metaplastic tumor is another extremely rare BC defined by the histological presence of at least two cell types, typically epithelial and mesenchymal components. This variant shows a TNBC phenotype with more aggressive behavior, less chemosensitivity, and a worse prognosis in comparison to other BC types (9). Based on the Surveillance, Epidemiology, and End Results (SEER) database and cases from the Union Hospital of Fujian Medical University, Zheng et al. analyzed prognostic factors (age, T stage, N stage, M stage, surgery, and radiotherapy) and constructed a nomogram to provide more accurate individualized survival analyses for patients with this rare histotype. Male BC is a seldom-occurring condition, accounting for less than 1% of all malignancies in men and less than 1% of malignant breast tumors. Due to the absence of established treatment guidelines, patients with BC are currently managed similarly to the female population. Nevertheless, male BC exhibits different characteristics and clinical behavior compared to its female counterpart, highlighting the need for a unique predictive model to develop a personalized therapeutic approach (10). To this end, Wen et al. developed a prediction model based on univariate and multivariate logistic regression analyses. By extrapolating data from the SEER registry between 2010 and 2015 and cases from Fujian Medical University Union Hospital, the authors showed that the type of surgery, age, T and M status, histologic grade, expression of ER and HER2, and use of chemotherapy were predictors of male BC prognosis and used them to construct a nomogram that outperformed the AJCC staging system. 

Improved survival rates following cancer diagnosis have resulted in an increase in the occurrence of second primary cancers. While extensive research has been conducted on the risks of second primary malignancies in female BC patients over several decades, there is a notable lack of knowledge when it comes to second primary tumors in men (11). Huang et al. performed an analysis of data from 1,843 male patients with BC collected from the SEER database. They employed competing risk models and nomograms to create tools for predicting the probability of cancer-specific mortality and the development of second primary malignancies. According to their predictive model, factors such as older age at diagnosis, advanced TNM stage, lack of surgery and radiotherapy, a waiting time of more than one month before treatment initiation, and positive hormone receptor and HER2 status were associated with a less favorable prognosis in male BC patients. Furthermore, they developed an additional prediction model to assess the risk of second primary malignancies in male BC survivors. This model aims to facilitate risk-based follow-up and counseling.

Nearly 10% of breast cancers are related to the inheritance of damaged genes. The most common inherited gene mutations that increase the risk of BC are involved in the DNA repair pathway. In particular, genetic variants in Homologous Recombination Repair (HRR) genes, including BRCA1 and BRCA2, ATM, PALB2, and RAD51, play a critical role in BC inheritance and susceptibility (12). Yu and Wang’s meta-analysis focused on the relationship between polymorphisms in the HRR RAD51, G172T XRCC2, and XRCC3 genes and BC risk, showing an increased cancer risk associated with polymorphisms in the RAD51 genes which was significantly higher in the Arab population.

Moreover, homologous recombination deficiency confers increased sensitivity to PARPi and platinum (13). In order to assess the efficacy and safety of various pharmacotherapies for patients with metastatic, locally advanced, or recurrent BC carrying pathogenic BRCA1/BRCA2 variants, Zhu et al. conducted a network meta-analysis including nine randomized controlled trials (RCTs) with 1,912 participants. They demonstrated that, despite the increased occurrence of side effects, the most effective treatment combination for patients with advanced BC harboring germline BRCA variants was the use of PARP inhibitors alongside platinum-based chemotherapy.

Furthermore, the complex crosstalk between tumor cells and other cells in the microenvironment contributes to defining the tumor’s profile and behavior. Among these, tumor-infiltrating immune cells play two contrasting roles: they can protect against tumor progression by killing immunogenic neoplastic cells but, at the same time, they can also contribute to tumor escape and drug resistance by shaping tumor immunogenicity. Reactivation of the immune system using immune checkpoint inhibitors (ICIs) has emerged as a promising therapeutic strategy for many solid tumors and, more recently, for BC patients. BC has traditionally been considered an immunologically “cold” tumor with a low tumor mutational burden. However, among BC, TNBC and HER2+ subtypes exhibit certain indicators of immunogenicity, including Tumor Mutational Burden (TMB), high Tumor Infiltrating Lymphocytes (TILs), and expression of immunoinhibitory molecules. Preclinical studies demonstrating the enhanced immune-mediated effects of anti-HER2 monoclonal antibody therapy when combined with PD-1 antibodies, strongly support the addition of ICIs in HER2+ BC. Various immunotherapeutic strategies, including combinations of anti-HER2 therapy with ICIs and novel vaccines, are currently under investigation for the management of HER2+ BC (14). Nevertheless, none of these approaches has received regulatory approval to date. Padmanabhan et al. developed a mathematical model-based study demonstrating that the combination therapy of trastuzumab (anti-HER2 monoclonal antibody) and BMS-202 (anti-PD-1/PD-L1 small molecule inhibitor) significantly inhibits the growth of HER2+ BC cell lines, surpassing the efficacy of monotherapies, even in an immune cell-depleted environment. Results from *in vitro* monoculture experiments suggest that BMS-202 may suppress tumor growth not only by modulating the immune response but also by interfering with HER2+ BC growth signaling pathways. However, further studies are needed to demonstrate the potential interaction between PD-1/PD-L1 inhibitors and HER2 growth signaling pathways in BC.

In addition to genetic aberrations and the tumor microenvironment, environmental conditions, which are known to vary with changes in altitude, are relevant modulators of disease development and outcome (15). Chen et al. focused on BC patients at high altitudes who showed distinct characteristics in patient delay, BMI, tumor size, lymph node metastasis, and subtype distribution. This study highlights the complexity of factors influencing BC heterogeneity and suggests the need for a personalized therapeutic approach for patients living at high altitudes.

The prognosis of BC is influenced not only by the intrinsic characteristics of the tumor and its interactions with the microenvironment but also, particularly in the early stages, by the impact of surgical and radiotherapy (RT) treatments, along with patient adherence to medical therapy.

In the early stages, breast-conserving treatment or mastectomy are the surgical options. Given the increasing incidence of BC in young women and the limited evidence available regarding its management in this population (16), Pu et al. explored whether young patients (≤35 years old) might derive greater survival benefit from either breast-conserving surgery (BCS) or mastectomy. They performed a univariate and multivariate logistic regression analysis to identify independent factors influencing the benefit of BCS in young BC patients. According to the nomogram, among patients aged ≤35 years, those with older age, with lower T and N stages, and treated with postoperative RT without chemotherapy were more likely to benefit from BCS. These findings provide clinicians with guidance for decision-making.

Adjuvant RT after BCS for early-stage BC is considered the standard treatment because it improves the survival rate and reduces the risk of recurrence. The supine position has been widely used for radiotherapy in BC, but some evidence suggests better cosmetic outcomes and lower rates of late toxicity in the prone position (17). Gao et al. compared the prone and supine positions to assess differences in dose distribution and normal organ sparing when using VMAT in these two positions. In addition, they aimed to identify the biotype that derives the greatest benefit from RT administered in the prone position. The greatest benefit of the prone position was reported in patients with right-sided BC, those characterized by a drooping breast shape, a larger breast and cup size, and, in particular, a larger chest height dimension.

Adjuvant endocrine therapy (AET) is a mainstay of treatment in the management of women with HR+ tumors. However, the side effects of AET pose a significant challenge for BC survivors, leading to irregular adherence and treatment interruptions, which may have detrimental effects on their overall survival (18). The review by Huifang et al. focuses on the mechanism of poor adherence to endocrine therapy in BC patients. Clinical data show that the neuro-immuno-endocrine mechanisms play a decisive role in the occurrence of adverse reactions leading to poor compliance. The rapid decrease in estrogen levels triggered by AIs within a short timeframe intensifies sympathetic activity, thereby modulating the release of inflammatory factors by diverse immune cells. Therefore, gaining a deeper understanding of the potential mechanisms underlying poor adherence during treatment could reveal pharmacological targets and guide early clinical intervention, aiming to improve adherence and maximize the benefits for BC patients.

In *de novo* metastatic disease, which accounts for approximately 6% of metastatic BC, locoregional therapy (LRT) is controversial with inconsistent results from randomized control trials (RCTs) (19). In their review, Merloni et al. examine all available data and aim to identify a specific patient subgroup that may derive the greatest benefit from LRT for the primary tumor. Even if the majority of RCTs did not support LRT of the primary tumor, this conclusion should be interpreted with caution in view of the limitations identified including small sample sizes and the utilization of outdated systemic therapies. Conversely, the results of some retrospective studies and one Turkish randomized trial suggest that patients with oligometastatic, bone-only disease, and HR-positive disease may be the best candidates for LRT. In this context, biomarkers such as circulating tumor cells (CTCs) and circulating tumor DNA (ctDNA) may be useful to better predict the metastatic disease course. Therefore, considering the advances in systemic therapies and radiotherapeutic/surgical methods the authors suggest designing further randomized trials, in which a properly selected population, and new biomarkers are strongly encouraged.

In conclusion, our Research Topic offers a comprehensive overview of various aspects of BC heterogeneity to unravel the complexity of BC. These efforts aim to lay the foundation for more effective and personalized diagnostic and therapeutic approaches. Continued research in this area is crucial, as it has the potential to guide future cancer therapy and ultimately improve outcomes.

## Author contributions

FC: Writing – original draft. CS: Writing – review & editing. MO: Writing – review & editing. BP: Writing – review & editing. AD: Writing – review & editing. MB: Writing – review & editing.
